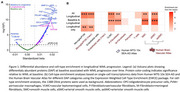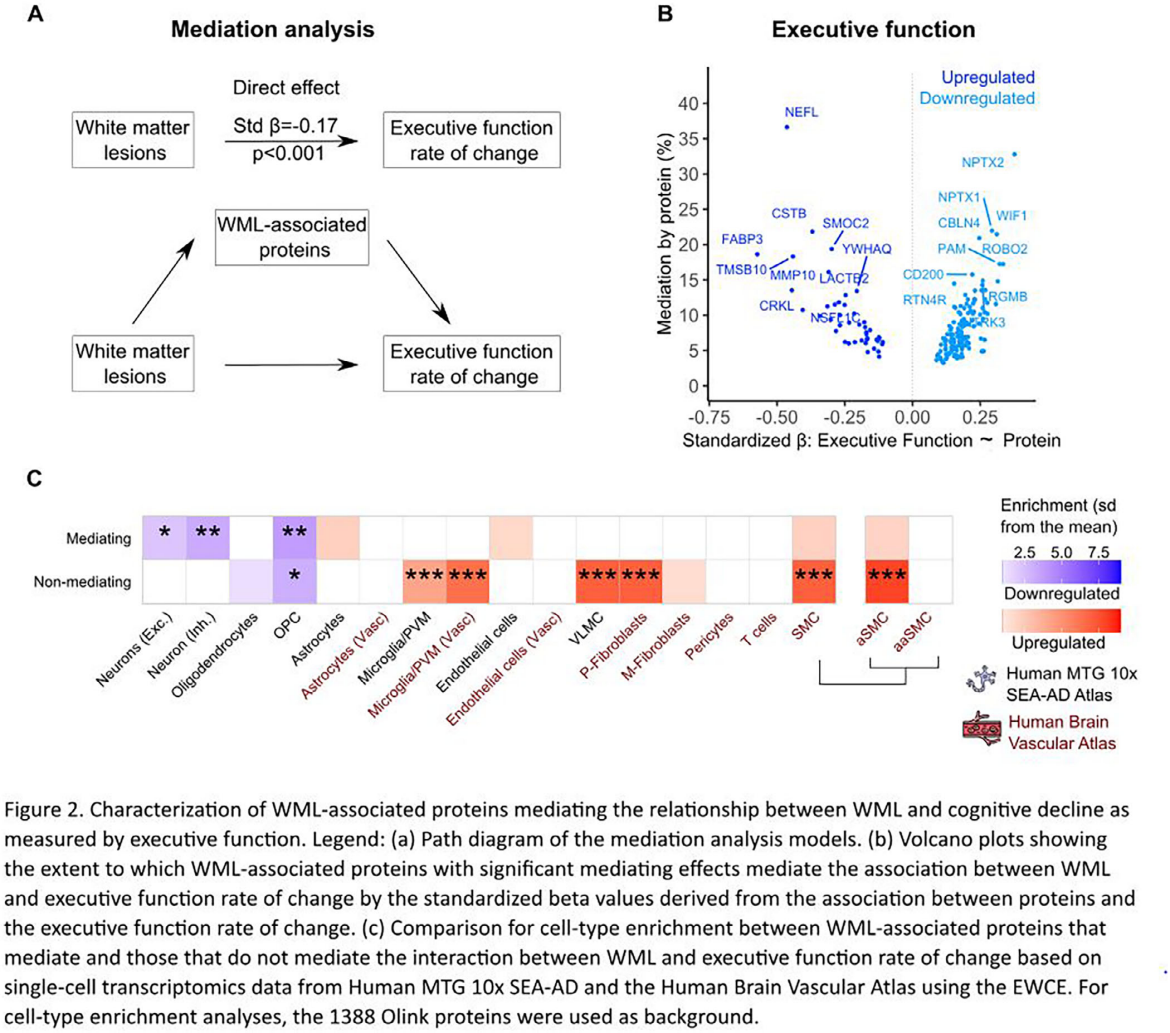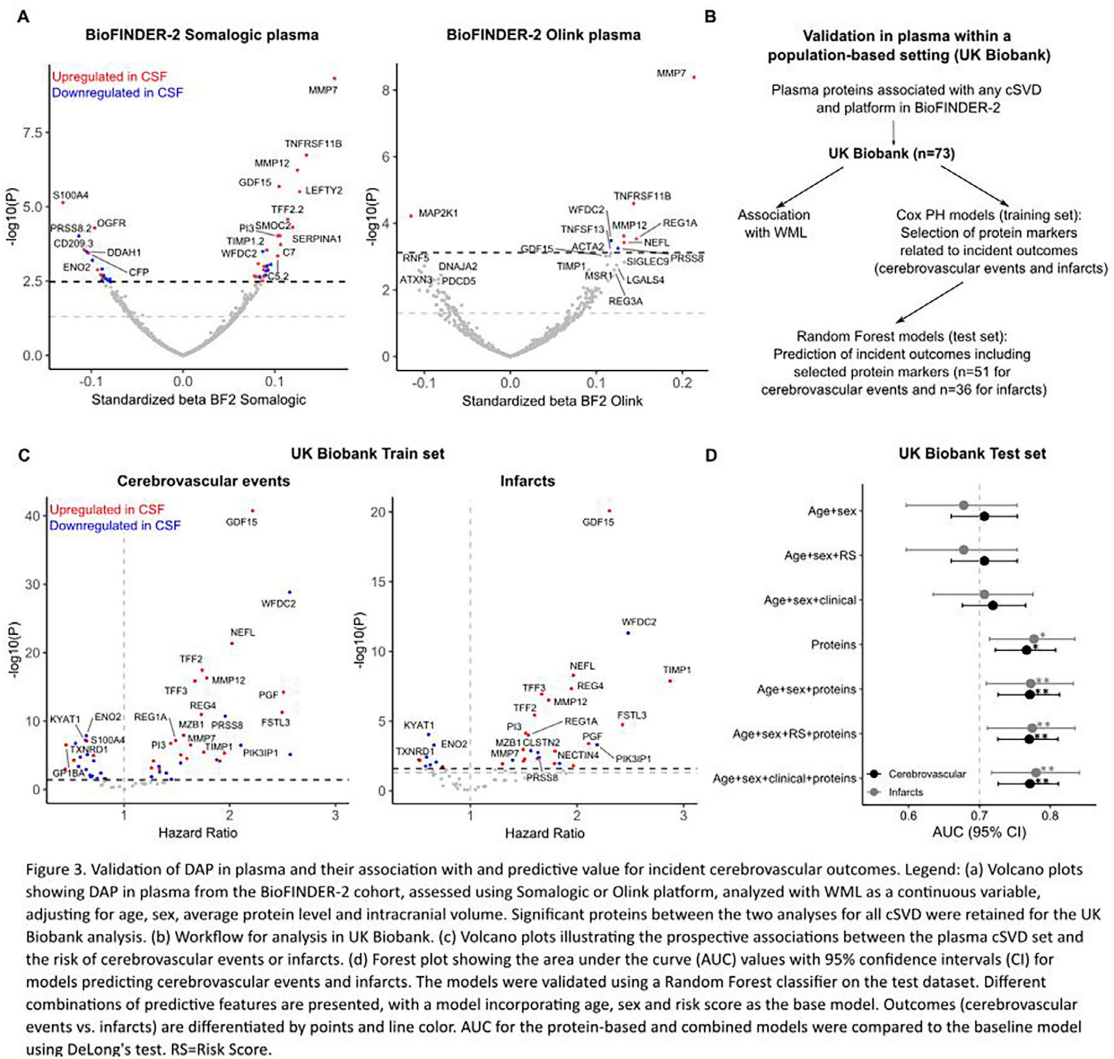# CSF and plasma proteomic insights into the pathophysiology of cerebral small vessel disease and cerebrovascular event risk prediction

**DOI:** 10.1002/alz70856_098830

**Published:** 2025-12-24

**Authors:** Ines Hristovska, Alexa Pichet Binette, Atul Kumar, Malin Wennström, Chris Gaiteri, Linda Karlsson, Olof Strandberg, Shorena Janelidze, Danielle van Westen, Erik Stomrud, Sebastian Palmqvist, Rik Ossenkoppele, Niklas Mattsson‐Carlgren, Jacob W. Vogel, Oskar Hansson

**Affiliations:** ^1^ Clinical Memory Research Unit, Department of Clinical Sciences, Lund University, Lund, Sweden; ^2^ Department of Physiology and Pharmacology, Université de Montréal, Montréal, QC, Canada; ^3^ Clinical Memory Research Unit, Department of Clinical Sciences Malmö, Lund University, MOntreal, QC, Canada; ^4^ Centre de recherche de l'institut universitaire de gériatrie de Montréal (CRIUGM), Montréal, QC, Canada; ^5^ Cognitive Disorder Research Unit, Department of Clinical Sciences Malmö, Lund University, 214 28 Malmö, Sweden, Malmö, skåne, Sweden; ^6^ Rush Alzheimer's Disease Center, Rush University Medical Center, Chicago, IL, USA; ^7^ Department of Psychiatry, Upstate Medical University, Syracuse, NY, USA; ^8^ Clinical Memory Research Unit, Department of Clinical Sciences Malmö, Faculty of Medicine, Lund University, Lund, Sweden; ^9^ Clinical Memory Research Unit, Department of Clinical Sciences Malmö, Faculty of Medicine, Lund University, Sweden, Lund, Sweden; ^10^ Imaging and Function, Skåne University Hospital, Lund, Sweden; ^11^ Diagnostic Radiology, Department of Clinical Sciences, Lund University, Lund, Sweden; ^12^ Memory Clinic, Skåne University Hospital, Malmö, Skåne, Sweden; ^13^ Clinical Memory Research Unit, Department of Clinical Sciences Malmö, Lund University, Lund, Sweden; ^14^ Amsterdam Neuroscience, Neurodegeneration., Amsterdam, Netherlands; ^15^ Alzheimer Center Amsterdam, Neurology, Vrije Universiteit Amsterdam, Amsterdam UMC location VUmc, Amsterdam, Netherlands; ^16^ Clinical Memory Research Unit, Lund University, Malmö, Skåne, Sweden; ^17^ Department of Neurology, Skåne University Hospital, Lund, Sweden; ^18^ Wallenberg Center for Molecular Medicine, Lund University, Lund, Sweden; ^19^ Department of Clinical Sciences Malmö, SciLifeLab, Lund University, Lund, Sweden; ^20^ Department of Clinical Sciences Malmö, Faculty of Medicine, SciLifeLab, Lund University, Lund, Sweden; ^21^ Memory Clinic, Skåne University Hospital, Malmö, Sweden

## Abstract

**Background:**

Cerebral small vessel disease (cSVD) is the leading vascular contributor to dementia and stroke, with white matter lesions (WML) as the most common manifestation. Despite its prevalence, the underlying pathophysiology remains poorly understood, and reliable plasma biomarkers are lacking. Leveraging multi‐omics approach, we aimed to: 1) explore the mechanisms underlying WML progression and cognitive decline, and 2) validate CSF‐identified biomarkers in plasma across cSVD manifestations and assess their potential to predict future cerebrovascular events.

**Method:**

We analyzed 1,388 CSF proteins using Olink in the Swedish BioFINDER‐2 cohort (*n* = 1,670). Differential protein abundance for cSVD manifestations (WML, microbleeds and infarcts) was evaluated using linear models, adjusting for age, sex, average protein levels and intracranial volume, if applicable. We used linear mixed‐effects models to identify proteins associated with WML progression and mediation analysis to determine WML‐associated DAP contributing to cognitive decline over six years. Plasma proteomics was assessed using SOMAscan7k (*n* = 1,599) and Olink (*n* = 694) in BioFINDER‐2, with DAP analysis, as previously described, focusing on cSVD‐associated proteins identified from CSF. In UK Biobank, we assessed cSVD‐associated plasma proteins identified in BioFINDER‐2 by examining their association with 5‐year risk of cerebrovascular outcomes using Cox proportional hazards models and evaluated their predictive utility with a Random Forest classifier (*n* = 51,606).

**Result:**

While many proteins were associated with baseline and WML progression, we identified a subset uniquely associated with WML progression, enriched in microglial and macrophage populations (Figure 1). The link between WML and cognitive decline was partly mediated by neuronal and OPC‐associated proteins (Figure 2). Key cSVD‐associated proteins in CSF, including MMP7, MMP12, TNFRSF11B and GDF15, were validated in plasma (Figure 3A). Using UK Biobank as a population‐based setting (Figure 3B), we identified a subset of cSVD‐associated plasma proteins related to the risk of cerebrovascular events (Figure 3C), which significantly improved 5‐year risk stratification (Figure 3D), surpassing models based on age, sex, and stroke risk score.

**Conclusion:**

Our findings reveal proteins linked to WML progression and cognitive decline, advancing the understanding of cSVD pathophysiology. Validation of key proteomic markers in plasma and demonstration of their predictive value for cerebrovascular outcomes highlight their potential for early risk stratification and personalized prevention.